# Suture tape reinforcement of hamstring tendon graft reduces postoperative knee laxity after primary ACL reconstruction

**DOI:** 10.1186/s40634-022-00454-2

**Published:** 2022-02-23

**Authors:** Christoffer von Essen, Vasileios Sarakatsianos, Riccardo Cristiani, Anders Stålman

**Affiliations:** 1 Department of Molecular Medicine and Surgery, Stockholm Sports Trauma Research Center, Karolinska Institutet, Stockholm, Sweden; 2 Capio Artro Clinic, FIFA Medical Centre of Excellence, Sophiahemmet Hospital, Valhallavägen 91, 11486 Stockholm, Sweden

**Keywords:** ACL, ACL reconstruction, suture tape reinforcement

## Abstract

**Purpose:**

To evaluate and compare subjective and objective knee outcomes following hamstring tendon (HT) and quadriceps tendon (QT) anterior cruciate ligament reconstruction (ACLR) with or without suture tape (ST) reinforcement. It was hypothesized that the addition of an intra-articular synthetic augmentation with a ST would reduce postoperative knee laxity and graft ruptures after ACLR.

**Methods:**

A 1:1 matched-cohort comparison of patients who underwent HT and QT autograft ACLR with or without ST reinforcement was performed. Patients with ST reinforcement were consecutively assigned to the study groups until a number of 20 in each group was achieved. Medical records were reviewed for demographic characteristics and additional injuries. Laxity measurements with KT-1000, strength measurements and physical examination findings were collected both preoperatively and at 6 months and patient reported outcome (PRO) scores were collected both preoperatively and at 12 months, and comparison was made HT vs HT + ST and QT vs QT + ST. Reoperations and re-ruptures were recorded during the 24-month follow-up period.

**Results:**

Overall, 80 patients who underwent ACLR were included. Patients with HT + ST had significant less laxity postoperatively compared to HT at 6 months, 1.9 vs 0.8 mm, *p* < 0.05. No differences were found between the QT and QT + ST group. At 6 weeks patients treated with ST, both QT and HT, had a significant deficit in flexion compared to those without ST. However, this resolved at 6 months. There were no significant differences between HT + ST vs HT, or QT + ST vs QT, regarding postoperative PROs or strength measurements. Furthermore, the incidence of subsequent surgery and graft rupture was not significantly different between the groups.

**Conclusion:**

ACLR with HT + ST reduces laxity at 6 months compared to ACLR without ST, a difference not seen when ACLR was performed using QT with or without ST. No other differences were seen between the two techniques comparing subjective and objective findings.

**Level of evidence:**

Level III.

## Introduction

Anterior cruciate ligament (ACL) reconstruction (ACLR) is a common orthopedic procedure, aiming at reducing knee instability. Although ACLR techniques have developed over the years and are today performed with an anteromedial femoral drilling technique, allowing a more anatomical positioning of the graft, the risk for subsequent surgery is still high [1, 2]. An ACL graft tear is detrimental to patient outcomes following ACLR. Systematic reviews indicate that one can expect a failure rate between 3.5 and 7% of autografts, increasing up to 10–28% in a high-risk population [3–6]. The rates of ACL revision surgery have been reported at 2–3% within the first two to three years and an overall revision rate of approximately 4% is reported in the Swedish Knee Ligament Registry (SKLR). Furthermore, it is evident that the outcome after revision ACLR is inferior to that of a primary ACLR [7–11].

In Sweden, a hamstring graft (HT) is utilized in more than 85% of ACLRs, but quadriceps graft (QT) is gaining popularity [12]*.* Proponents of HT have reported minor donor-site morbidity, good tissue strength and easy access [13, 14], but with HT there is a high variability in the diameter of the graft received and a smaller diameter has been associated with a higher risk of graft failure [15, 16]. HTs are also associated with an increased risk of increased knee laxity after primary ACLR [17, 18]. The QT has shown to be a good alternative, with comparable outcomes and failure rates as well as the possibility to customize graft diameter [13, 19–21].

Graft augmentation was first introduced in the 80s to increase tensile strength of the graft, but outcome data showed high failure rate and complications [22, 23]. However, more recently reinforcement with suture tape (ST) has been described to improve clinical outcomes and is thought to support the graft after ACLR [24, 25]. ST reinforcement has been used in various ligament repairs and reconstructions [26–29]. The ST is composed of an ultrahigh-molecular-weight polyethylene/polyester tape that is collagen coated to increase tissue integration and proposed advantages include protection of the graft during the proliferation, maturation, and ligamentization phases of healing [24, 25].

Only a few studies have compared HT ACLR with or without ST reinforcement and none using QT [30, 31].

The optimal surgical methods, graft choice and rehabilitation for ACLR have not yet been established. Rehabilitation must be fine-tuned in order to limit strain on the graft during graft incorporation and maturation. Although ACLR allows return to sports for the majority of patients [32], there is a risk of suffering a new injury or developing persistent laxity [33]. As such there remains potential for improvements in the treatment of these patients.

The purpose of this study was to compare subjective and objective outcomes for patients undergoing ACLR with either a HT or QT graft, with or without complementary ST. It was hypothesized that the addition of an intra-articular synthetic augmentation of the graft with a ST would reduce the risk of residual laxity and graft rupture after ACLR.

## Material and method

Ethical approval for this study was obtained from the regional ethics committee, Karolinska Institutet 2017–401/31.

Patients presenting with an isolated ACL deficiency at the orthopedic outpatient clinic were assessed and received standardized written and oral information about the trial. Informed consent was obtained from each patient prior to participation to the study. Inclusion criteria were: Physical examination and magnetic resonance imaging (MRI) showing ACL injury, age between 18 and 40 years, active participation in pivoting sports and an ambition to return to the same sport. Exclusion criteria were: (1) previous ACL injuries to the ipsi- or contralateral knee, (2) multi-ligament injuries.

After patient and surgeon agreed on ACLR and graft choice, the patients were consecutively assigned to the study groups until a number of 20 patients with HT graft and ST and 20 patients with QT and ST were achieved.

**Fig. 1:**
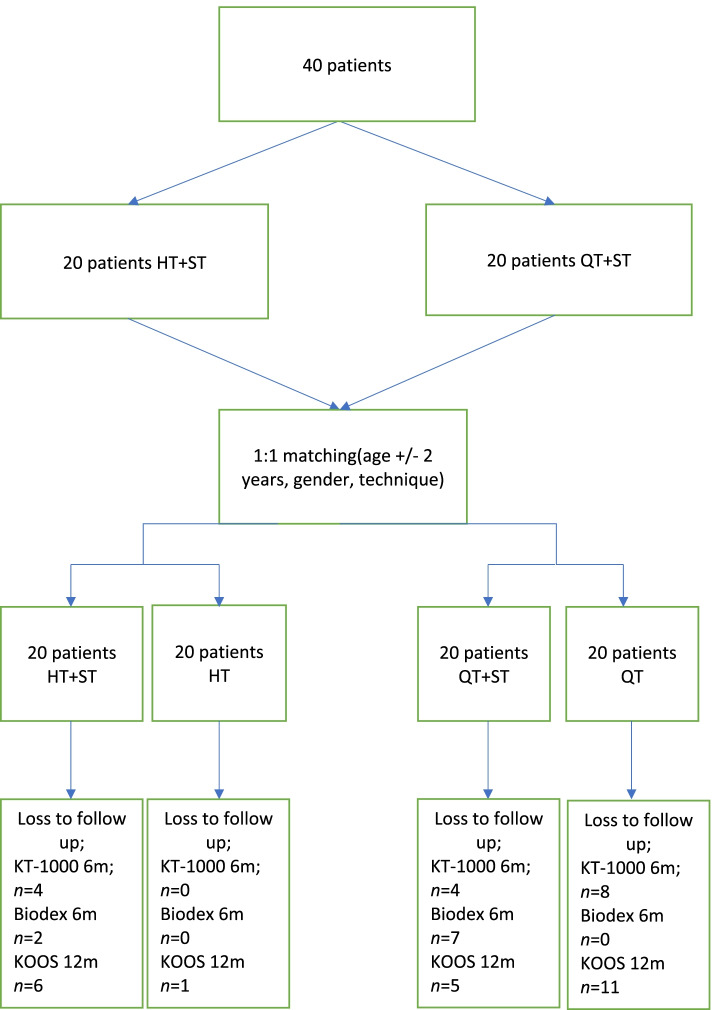
Flow chart. KOOS, Knee injury and Osteoarthritis Outcome Score; HT, hamstring tendon; ST, suture tape; QT,quadriceps tendon

The patients were then matched in a 1:1 ratio to a control group (age +/− 2 years, gender) consisting of patients who underwent surgery at the clinic during the same time frame and with the same surgical technique, apart from the ST reinforcement, Fig. 1.

### Surgical technique

Patients included in the study underwent anatomical single-bundle ACLR with autologous HT graft or mid-portion, full-thickness, all-soft tissue QT graft, with or without the addition of an independent ST (fiber tape 2.0 mm, AR-7237, Arthrex, Naples, FL). Surgery was performed by one of two experienced knee surgeons.

The HT graft was harvested through a short, anteromedial oblique incision, a semitendinosus autograft was used in all cases and all grafts were between 8 and 10 mm in diameter as a quadruple.

The QT was harvested at full depth from a position slightly lateral to the central part of the patella. A graft with one-centimeter width was harvested with a length of seven centimeters and cut with a quad tendon graft cutting guide and quad tendon stripper/cutter (Arthrex Inc., Naples, FL) without the use of a bone-plug.

The graft ends were prepared using a SpeedWhip ripstop technique with No. 2 FiberLoop with a FiberTag (FiberLoop® with FiberTag™ Suture, Arthrex, Inc., Naples, FL). Graft diameter was 10 mm.

Suspensory fixation was used on both sides, a TightRope® RT (Arthrex, Inc., Naples, FL) was used on the femoral side, and a TightRope® ABS (Arthrex, Inc., Naples, FL) with a 14- or 20-mm button on the tibial side.

**Fig. 2:**
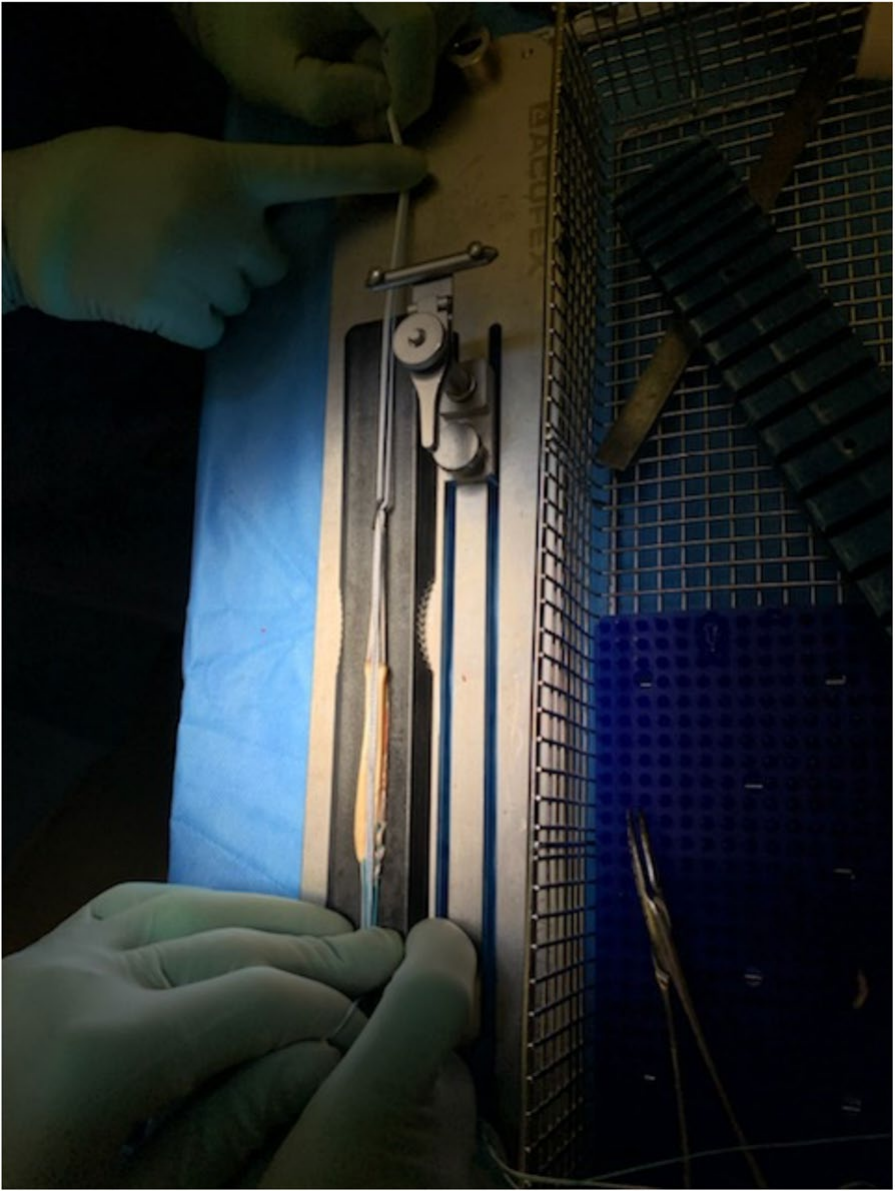
Illustration of the reinforcement technique with FiberTape leading to a combined load distribution with the independent fixed FiberTape as a protective “safety belt”

**Fig. 3:**
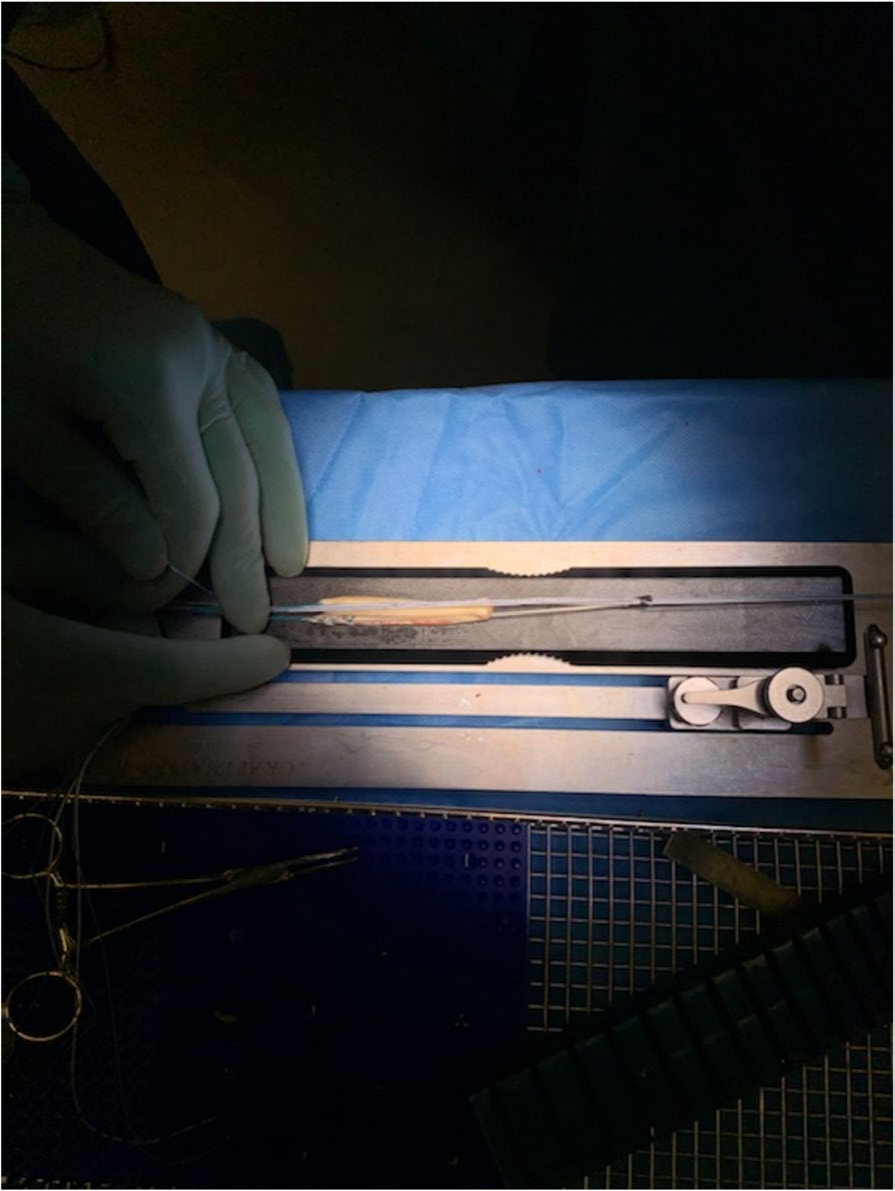
Illustration of the reinforcement technique with FiberTape leading to a combined load distribution with the independent fixed FiberTape as a protective “safety belt”

**Fig. 4:**
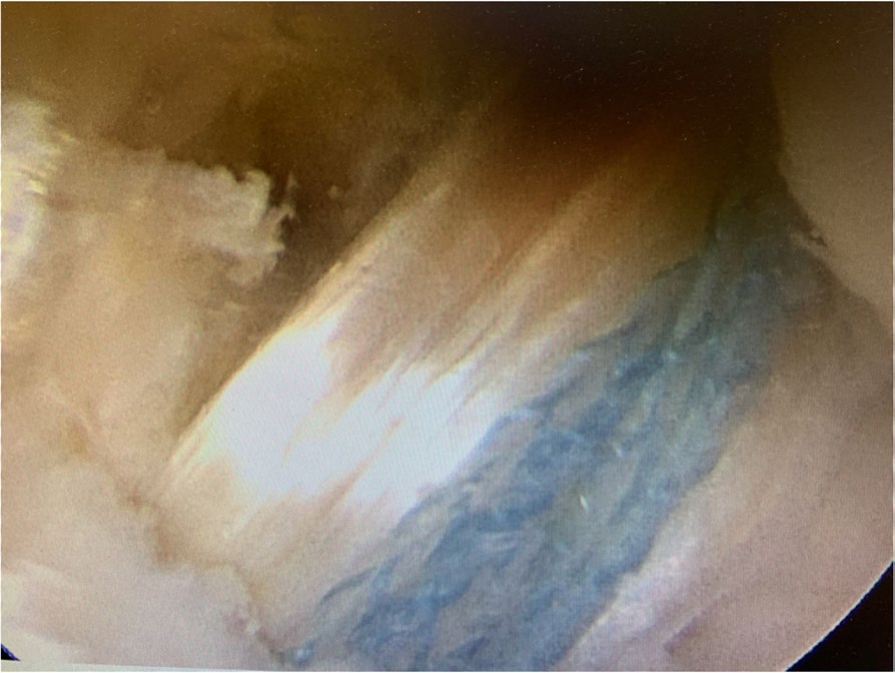
Illustration of the reinforcement technique with FiberTape leading to a combined load distribution with the independent fixed FiberTape as a protective “safety belt”

For the ST-reinforced grafts, the reinforcement was done after graft preparation with a FiberTape® (fiber tape 2,0 mm, AR-7237, Arthrex, Naples, FL) intended to be an internalbrace™ (Arthrex, Inc., Naples, FL). The FiberTape® was passed through the proximal TightRope plate, Figs. 2, 3 and 4. The procedure was then similarly repeated at the distal end of the graft, with the exception that here a TightRope ABS button (Arthrex, Inc., Naples, FL) was used. The graft was placed in a graft tube (Arthrex Inc., Naples, FL) facilitating graft compression and shaping.

The femoral tunnel was drilled through the anteromedial portal in maximum knee flexion and was placed in the center of the native ACL footprint. For tibial tunnel placement, the ACL stump and the anterior horn of the lateral meniscus were used as landmarks.

The graft was first positioned in the femoral socket by tightening the Tightrope RT. A Tightrope ABS button was mounted on the ABS sling on the anterior tibia and the FiberTape was also passed through the button. The graft was fixed over the ABS button in 30 degrees flexion and the FiberTape - InternalBrace – sutures were tied over the tightrope button in full extension. Primary tension of the graft combined with a fibertape supposedly allows for creating a protective safety belt for soft tissues grafts [24]. Tension was done according to the surgeons preference and isometry of the construct. When tensioned in full extension, the movement will not be affected [34, 35] .

### Rehabilitation

The rehabilitation was standardized according to the clinic’s routine. Full weight bearing and full range of motion were allowed immediately postoperatively. Quadriceps strengthening was restricted to closed kinetic chain exercises during the first three months and thereafter rehabilitation was progressed with introduction of sport-specific exercises in order to enable return to the desired activity after 9 months and Biodex® testing showed 90% strength. If the menisci were in need of suturing, a brace was used with a fixed ROM 0–30° for 2 weeks, 0–60° for 2 weeks and 0–90° for another 2 weeks, full weight-bearing was permitted with the support of crutches during the first three weeks. Otherwise the same rehabilitation protocol was used.

### Patient evaluation

Demographics were obtained at baseline and included age, gender, injured side, time from injury to surgery and concomitant injuries.

The Knee injury and Osteoarthritis Outcome Score (KOOS) [36], was obtained preoperatively and at 12 months postoperatively. Preoperative physical examination included: measurement of range of motion (ROM), Lachmann test, anterior drawer test and pivot shift test. Grading of the ROM, Lachmann, anterior drawer test and pivot shift test was done in accordance to the International Knee Documentation Committee (IKDC) knee examination form [37].

The KT-1000 arthrometer (MEDmetric, Corp., San Diego, CA, USA), was used to evaluate anterior knee laxity preoperatively and 6 months postoperatively. A standard 30 lbs. force, corresponding to a 134-N anterior tibial load, at 20° of knee flexion, was applied. At least 3 measurements of each knee were made, and the median value was registered. Postoperative difference in displacement (side-to-side (STS) difference) values were stratified according to the IKDC knee examination form in three groups: ≤ 2, 3–5, and > 5 mm [37]. “Surgical failure” was defined as an STS difference greater than 5 mm (IKDC grade C and D).

Follow-up examinations at the clinic with visits to the surgeon and physiotherapist were performed at 6 weeks and 6 months postoperatively. At the follow up visits, patients underwent a full physical examination and at 6 months isokinetic strength was also measured with Biodex system 3 (Biodex Medical Systems, Shirley, New York, USA) [38].

Additionally, ACL graft failures, contralateral ACL ruptures and meniscal repair failures with necessity of revision meniscus surgery were recorded during the 24-month study period.

### Statistical analysis

For statistical analysis, SPSS® 25.0 (IBM SPSS Statistics, New York, USA) for Mac was used. Comparisons were made in pairs, HT vs. HT + ST and QT vs. QT + ST. To compare parametic and nonparametic variables between the groups the independent t-test and Mann-Whitney U test were used. Nominal variables were tested by the Pearson Chi-squared test. Paired-sampled t test was used for Longitudinal statistics for normally distributed scale variables. The significance level was set at *p* < 0.05.

To determine the effect size and the power of the study, a post hoc power analysis using G*Power 3.1.9.2 (Franz Paul, Kiel, Germany) was used. Based on laxity with KT1000 at 6 weeks follow-up, the analysis revealed that for HT, a sample size of 20 patients in each group would yield a power of 61% to detect a 5% significance level in laxity and an effect size of 0.73 was obtained. The total sample size needed for a power 80% would be 62 patients, 31 in each group. For QT, a sample size of 20 patients in each group would yield a power of 5% to detect a 5% significance level in laxity and an effect size of 0.06 was obtained. The total sample size needed for a power 80% with QT would be 8140 patients, 4070 in each group.

## Results

Demographic data of the study groups are displayed in Table 1. No significant differences were present among the groups.

**Table 1:** Patient characteristics

	HT + ST *n* = 20	HT Control *n* = 20	*p*-value	QT + ST *n* = 20	QT Control *n* = 20	*p*-value
Age, mean (range)	28.7 (17–42)	29.1 (17–44)	n.s.	29.6 (18–48)	29.2 (18–48)	n.s.
Sex						
Male	11 (55)	11 (55)		12 (60)	12 (60)	
Female	9 (45)	9 (45)		8 (40)	8 (40)	
Medial meniscus resection	2 (10)	2 (10)	n.s	2 (10)	2 (10)	n.s
Medial meniscus repair	7 (35)	4 (20)	n.s	3 (15)	2 (10)	n.s
Lateral meniscus resection	6 (30)	4 (20)	n.s	1 (5)	3 (15)	n.s
Lateral meniscus repair	1 (5)	2 (10)	n.s	1 (5)	1 (5)	n.s
Cartilage injury	1 (5)	2 (10)	n.s	3/20	4 (20)	n.s

Data are reported as n (%), unless otherwise indicated

*HT* Hamstring tendon, *ST* Suture tape, *QT* quadriceps tendon

### Laxity

**Fig. 5:**
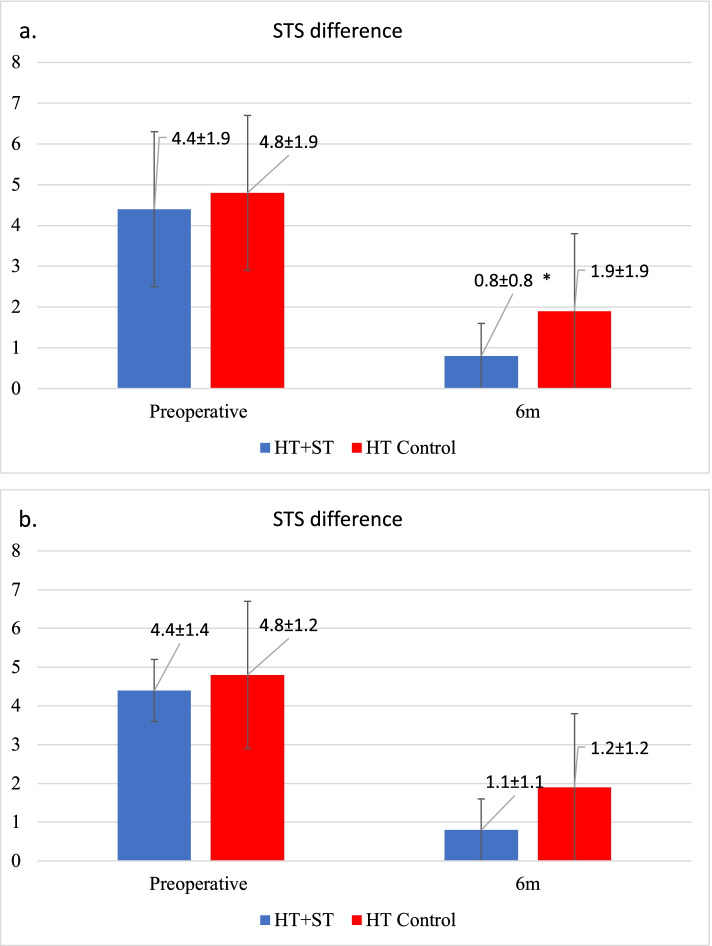
**a-b** Mean anterior ± SD STS difference, Values are displayed in mm. HT, hamstring tendon; ST, suture tape; QT, quadriceps tendon. * Statistically significant

The mean preoperative and postoperative anterior STS difference showed that the HT with ST had significantly less laxity postoperatively compared with HT alone, no difference was found between the QT with or without ST, Fig. 5a-b. No group had an STS difference greater than 5 mm (surgical failure). Almost 70% of the patients had less than 2 mm STS difference in the HT + ST group, Table 2.

**Table 2:** Stratified KT-1000 values according to IKDC at 6 m

	HT ST *n* = 16	HT Control *n* = 20	*p*-value	QT ST *n* = 16	QT Control *n* = 12	*p*-value
< 2 mm	11 (69)	8 (40)	n.s.	9 (56)	6 (50)	n.s.
2–5 mm	5 (31)	12 (60)	n.s.	7 (44)	6 (50)	n.s.
> 5 mm	0	0		0	0	

Data are reported as n (%), unless otherwise indicated

*IKDC* The International Knee Documentation, *HT* Hamstring tendon, *ST* Suture tape, *QT* quadriceps tendon

### Objective results

At 6 weeks patients treated with both QT + ST and HT + ST had a significant deficit in flexion. This difference resolved at 6 months, Table 3.

**Table 3:** Patient objective outcome and muscle strength

	HT + ST *n* = 20	HT control *n* = 20	*p*-value	QT + ST *n* = 20	QT control *n* = 20	*p*-value
ROM^a^						
Loss of extension						
Preop	0	0	n.s.	0	0	n.s.
6w	6 (30)	4 (20)	n.s.	4 (20)	2 (10)	n.s.
6 m	0	0	n.s.	0	0	n.s.
Loss of flexion						
Preop	0	0	n.s.	0	0	n.s.
6w	11 (55)	4 (20)	0.029	10 (50)	4 (20)	0.046
6 m	1 (5)	2 (10)	n.s.	1 (5)	1 (5)	n.s.
Hydrops^b^						
Preop	0	0	n.s.	0	0	n.s.
6w	12 (60)	9 (45)	n.s.	12 (60)	8 (40)	n.s.
6 m	2 (10)	1 (5)	n.s.	1 (5)	1 (5)	n.s.
Clinical laxity^c^ at 6 m						
Lachman	4 (20)	3 (15)	n.s.	3 (15)	3 (15)	n.s.
Pivot Shift	0	0	n.s.	0	0	n.s.
Biodex 6 m LSI ≥ 90%^d^						
Quadriceps strength	5 (28)	5 (25)	n.s.	1 (8)	1 (5)	n.s.
Hamstring strength	4 (22)	6 (30)	n.s.	10 (77)	12 (60)	n.s.

Values are displayed as number and (%) percentage

*HT* Hamstring tendon, *ST* Suture tape, *QT* Quadriceps tendon

^a^ROM at 6 weeks and 6 months is compared to pre-op values in the operated knee

^b^Hydrops according to IKDC

^c^Assesses stability of knee at rest result range from 0 (normal stability) to 1 (increased instability)

^d^ The limb symmetry indexes (LSIs) of the peak quadriceps and hamstring torque were calculated as [involved limb/uninvolved limb × 100] for each test. The achievement of a symmetrical isokinetic muscle strength was defined as performing at least 90% of the contralateral leg (LSI ≥ 90%)

No differences were found in hydrops, clinical laxity or in strength measurements between the groups, Table 3.

### Patient reported outcomes

**Fig. 6:**
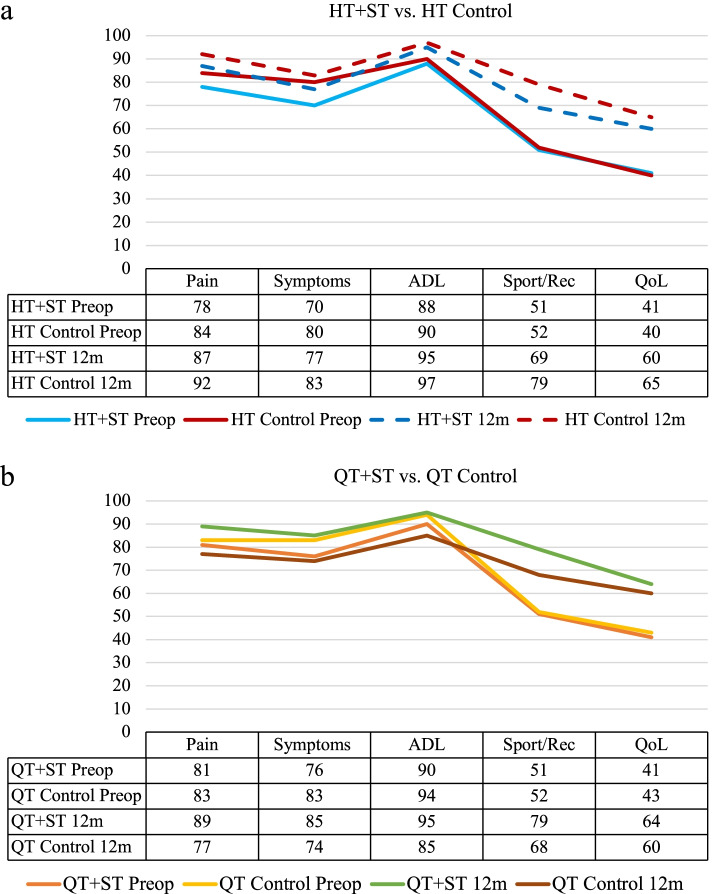
**a-b**, Mean KOOS score pre and postoperative at 12 m for the groups. KOOS, Knee injury and Osteoarthritis Outcome Score; HT, hamstring tendon; ST, suture tape; QT,quadriceps tendon

Figure 6 shows KOOS scores preoperatively and at 12 months postoperatively. There were no significant differences between the groups. Scores significantly increased from preoperatively to 12 months.

### Graft rupture and subsequent surgery

All patients with graft rupture reported a new trauma. The incidence of subsequent surgery and graft rupture did not differ significantly between the groups, Table 4. In the HT + ST group, 2 patients underwent synovectomy due to pain and flexion deficit. In the QT control group 1 patient had a graft rupture after a new trauma 18 months after the index surgery, however, no further surgery was performed. In the QT + ST group, 2 patients experienced discomfort and one had a slight flexion deficit, − 10 degrees, diagnostic arthroscopies were performed in both cases without any findings. After surgeries, the discomfort subsided. One patient suffered a new trauma during snowboarding. There was a small new cartilage injury on the lateral femoral condyle, and the patient underwent microfracture surgery. The graft was intact, but the ST was torn.

**Table 4:** Re-operations and re-rupture. Two years follow-up

	HT + ST *n* = 20	HT control *n* = 20	*p*-value	QT + ST *n* = 20	QT control *n* = 20	*p*-value
Re-operation^a^	5 (25)	4 (20)	n.s.	5 (25)	4 (20)	n.s.
Confirmed ACL re-rupture	1 (5)	0	n.s.	0	2 (10)	n.s.
Synovectomy	2 (10)	0		0	0	
Revision ACLR	1 (5)	0		0	1 (5)	
Cyclops	1 (5)	2 (10)		0	1 (5)	
Meniscal procedure	1 (5)	2 (10)		0	2 (10)	
Notchplasty	0	0		1 (5)	0	
Cartilage procedure	0	0		2 (10)	0	
Diagnostic arthroscopy	0	0		2 (10)	0	

Values are displayed as number and (%) percentage

*HT* Hamstring tendon, *ST* Suture tape, *QT* Quadriceps tendon

^a^Number of patients, one patient can have more than one reoperation

## Discussion

The principal finding in this matched-cohort comparison pilot study was that the use of an HT graft with ST reinforcement significantly reduced laxity with KT-1000 at 6 months compared with HT alone. However, at 6 months no differences in laxity were seen with the QT + ST compared to QT. Further, this study did not identify significant differences in re-rupture and reoperation rates, objective measurements and PROs between the groups.

The results of this study showed neither a higher complication rate nor hydrops in the cohort with ST compared to the control cohort. This is in contrast with the results reported in the 1980s, when the use of augmentation led to high rates of failure and noninfectious knee synovitis/effusion [22]. However, more recent studies have shown similar results regarding synovitis [30, 31]. The re-rupture rates were also low after 2 years.

One of the primary goals of ST reinforcement in ACLR is protection of the graft, especially early in the healing process. The ST is supposed to act as a safety belt that supports the graft during the remodeling phase offering protection from high strains [39]. There is a high risk of subsequent injuries when returning to sport after ACLR [40, 41] suggesting that the reconstructed graft cannot tolerate the necessary forces. Graft ligamentization is a slow process and it is not exactly known how long it takes before the graft resembles the properties of the native ACL [42]. Biomechanical studies on ST reinforcement showed reduced elongation of the graft (24,35), which can also be seen in this study as the HT + ST graft showed significant reduced laxity compared to the control group [24, 43]. The surgical technique of independent reinforcement with a ST to the graft is not technically demanding and can be done with minimal extra time during graft preparation. It does, however, have the potential to over constrain the knee, if the ST is tensioned too tightly. This could result in stiffness or loss of ROM and possibly also interfere with the incorporation of the graft. Applying the ST separately and having separate fixation allows the graft to function independantly within a normal ROM, thus avoiding overconstraint of the construct [34, 35]. This can be seen as a safety-belt that doesn’t inhibit the graft healing process but helps in avoiding unintended excessive graft strain during rehabilitation or return to sport. In this study, both groups with ST had a significant loss of flexion at 6 weeks postoperatively compared to the control group. Possibly the graft with ST needed more time to adapt. It is possible that applying a ST can give a temporary over constraint of the knee. This loss of motion did, however, resolve at 6 months.

There were no differences in postoperative KOOS subscales scores at 1 year. However, there were significant improvements for all groups on all subscales from preoperative level.

The main strength of this study was that we could show that the technique is feasible without any” red flags” that previously have been described with other augmentation techniques [22].

Being a pilot study there are limitations. There was no randomization between the groups, neither were the patients nor surgeons blinded to the treatment and reported outcomes could have been subject to bias. There is a limitation that the KOOS values are only at one-year follow up, even though KOOS results are shown to be equivalent between 1 and 2 years in register data [44]. Another limitation is that the graft tension was not standardized.

A post hoc analysis revealed a high effect size for the ACLR with HT graft and ST, but for the ACLR with QT graft and ST the effect size was very low. The results from the post hoc analysis revealed that the total sample size needed to detect a difference in laxity for the HT + ST was 62 patients if desired power is set at 80%.

The results from this pilot study are important and show that further studies are feasible, and that there are potential benefits regarding laxity, foremost with HT + ST.

## Conclusions

Anterior cruciate ligament reconstruction with HT + ST reduces laxity compared to ACLR without ST, a difference not seen when ACLR was performed with QT + ST compared without ST. No other differences were seen between the two techniques comparing subjective and objective findings.

## Data Availability

The data presented in this study are available on request from the corresponding author.
